# Evaluation of Medication Errors in a Tertiary Care Hospital of a Low- to Middle-Income Country

**DOI:** 10.7759/cureus.16769

**Published:** 2021-07-31

**Authors:** Ashwaghosha Parthasarathi, Rahul Puvvada, Himanshu Patel, Pooja Bhandari, Sagar Nagpal

**Affiliations:** 1 Epidemiology and Public Health, Allergy, Asthma and Chest Centre, Mysore, IND; 2 Physiology, Anatomy, and Microbiology, College of Science, Health and Engineering, La Trobe University, Melbourne, AUS; 3 Pharmacology and Therapeutics, International Society of Oncology Pharmacy Practitioners, North Vancouver, CAN; 4 Public Health, Madhavnagar Government Hospital, Ujjain, IND; 5 Internal Medicine, Erie County Medical Center, Buffalo, USA

**Keywords:** medication errors, patient safety, causes of medication errors, medication safety, prescribing errors, dispensing errors, administration errors

## Abstract

Introduction

Medication errors (MEs) are a major public health concern as they are detrimental to patient safety, compromise patients' confidence in the healthcare system, increase healthcare costs, and adversely affect the patient's quality of life. This is especially true in low to middle-income countries where the significance of MEs is largely undervalued. This study aims to investigate the prevalence of MEs and analyze the causes, medicines involved, reporting, and severity of MEs in a tertiary care setting.

Methods

A prospective observational study was conducted from March 2020 to February 2021 in a tertiary care teaching hospital in South India. The data was collected after reviewing patient medical records, by interviewing patients and healthcare professionals. National Coordinating Council for Medication Error Reporting and Prevention (NCC MERP) index was used to evaluate MEs.

Results

A total of 557 MEs were identified from 3798 patients with a prevalence of 14.6%. Prescribing errors were the most commonly observed ME followed by errors related to documentation of medical records, administration-related errors, and dispensing errors. Lack of time for documentation of medication records, shift change and work overload were common causes of MEs. The majority of MEs were category A and B of the NCC MERP severity index.

Conclusion

Antibiotics and proton pump inhibitors were the most common medicines involved in MEs. Prescribing and documentation errors were most prevalent. Implementation of systems like strict adherence to treatment guidelines, computerized provider order entry (CPOE), barcode medication administration, and closed-loop electronic medication management systems may greatly help reduce MEs. All healthcare institutions should undertake routine audits to determine the prevalence and causes of medication errors.

## Introduction

A medication error (ME) is deﬁned as “any preventable event that may cause or lead to inappropriate medication use or patient harm while the medication is in the control of the healthcare professional (HCP), patient, or consumer. Such events may be related to professional practice, healthcare products, procedures, and systems, including prescribing, order communication, product labeling, packaging, and nomenclature, compounding, dispensing, distribution, administration, education, monitoring, and use” [1].

MEs could occur at the level of prescribing, dispensing, or during administration. Hence, the problems and sources of MEs are multidisciplinary and multi-factorial [2]. Patients who are hospitalized are more likely to suffer from MEs, especially those patients who may be on polypharmacy, being treated under the supervision of multiple specialists, and having various co-morbid conditions [3].

The concept of medication errors (MEs) is not new. Its importance exists in the healthcare system globally. MEs are known to occur in any health care setting and are well documented in the published literature [4-6]. It is estimated that 7000 to 9000 people die of MEs annually in the United States alone [6]. Worldwide, billions of dollars are being spent managing MEs in all types of health care settings. Nearly two decades ago, researchers estimated that the annual cost of drug-related illness and death in the ambulatory care setting in the United States was approximately $76.6 billion [7]. The updated annual cost of drug-related morbidity and mortality from non-optimized medication use of $528.4 billion is 16% of total US health care expenditures compared to 8% and 13% in 1995 and 2008, respectively [8].

Regrettably, MEs are like an “Iceberg” as what we see is often a fractional part of what exists [9]. Many MEs remain concealed when the outcomes do not affect hospitalized patients. But, it is a significant threat to attaining the therapeutic goals by compromising patients' confidence in the healthcare system, increasing healthcare costs, and harming the patient's quality of life [5, 10, 11].

In this regard, on March 29, 2017, the World Health Organization (WHO) has launched “Medication Without Harm” as a theme for its third Global Patient Safety Challenge. The aim of this program is to reduce the severity of avoidable medication-related problems by half in the next five years [12].

But the scenario of MEs in developing countries such as India is still alarming and needs vigorous attention to protect health care consumers. Based on 2017 global health-related sustainable goals, India ranked 143 out of 184 countries [13]. The main causes of such a ranking come down to the lack of overall healthcare infrastructure, lack of a medically insured population with high out-of-pocket expenditure, and medication misadventure (adverse drug reactions i.e. ADRs and MEs) [14]. The quality of health services is a significant concern, with over-prescription of antibiotics, steroids, and delayed referrals being severe issues commonly encountered [15]. A recent cross-sectional study found that 77% of all reported medication errors occurred during the preparation and administration of medications with a majority of them were attributed to nurses, junior doctors, and pharmacists [16].

Considering the global scenario and Indian health care practices, there is a need to further evaluate MEs in the Indian health care system. This study aims to investigate the prevalence of MEs and analyze the causes, drugs involved, reporting, and severity of MEs in a tertiary care setting.

## Materials and methods

This prospective observational study was conducted from March 2020 to February 2021 in a tertiary care teaching hospital in Southern India. This study was approved by the Institutional Ethics Committee of SUIMS Institute of Medical Sciences and Research Center, Karnataka, India (Approval number: SUIMS/IEC/2017/ACA STUDY 04/ 2018-19). The study site was a 1200 bed multi-specialty hospital in the general medicine in-patient wards. Patient selection for the study was done in a "consecutive" manner i.e., all patients admitted to the wards during the study period. The patient population had a mixed patient population from different socio-economic backgrounds, with a majority of them from lower socio-economic status.

Data was collected on a designed data collection form by reviewing patients' medical records, interviewing patients, and interacting with concerned health care professionals (HCPs) by maintaining confidentiality to identify and confirm ME(s). The data was collected with the help of a multidisciplinary team which consisted of physicians, pharmacists, nurses, medical and pharmacy postgraduate students and interns. All the data collected was reviewed by the “Medication Error Review Committee” which consisted of a physician, pharmacist, and nursing in-charge as its members. The details collected included demographics of the patient, type of medication error, details of healthcare professional involved, the reason for the error, action is taken to resolve them, and outcome of error. All the identified MEs were reviewed and acknowledged by the medical investigator and then documented electronically. All MEs were evaluated further for their levels, types, causes, and its outcome.

The levels of identified MEs were determined as prescribing, documentation, administration, and dispensing error as suggested by National Coordinating Council for Medication Error Reporting and Prevention (NCC MERP) [1]. These MEs were classified into subtypes based on their nature, documentation, administration, and dispensing errors. These MEs were further divided into subtypes based on the nature of the occurrence.

After identifying MEs, an attempt was made to identify the causes of MEs. Based on the involvement of different HCPs and types of errors, causes were considered like knowledge-based errors, lack of attention, work overload, change in duty shifts. However, these causes are based on the perception of the investigator. The medications involved in MEs were classified based on their Anatomical Therapeutic Chemical (ATC) classification system. The severity of MEs was categorized based on the NCCMERP index for Categorizing Medication Errors. NCC MERP classifies the outcome of ME from Category A to Category I (Table 1).

**Table 1:** NCC MERP definition of a medication error and risk assessment index NCC MERP: National Coordinating Council for Medication Error Reporting and Prevention

Category	Description of category
No error	
A	Circumstances or events that have the capacity to cause error
Error, no harm	
B	An error occurred, but the medication did not reach the patient
C	An error occurred that reached the patient but did not cause patient harm
D	An error occurred that resulted in the need for increased patient monitoring but no patient harm
Error, harm	
E	An error occurred that resulted in the need for treatment or intervention and caused temporary patient harm
F	An error occurred that resulted in initial or prolonged hospitalization and caused temporary patient harm
G	An error occurred that resulted in permanent patient harm
H	An error occurred that resulted in a near-death event (e.g., anaphylaxis, cardiac arrest)
Error, death	
I	An error occurred that resulted in patient death

Statistical analysis was done by means of Jamovi (v1.6, The jamovi project). Continuous variables were presented as mean ± standard deviation while categorical variables were presented as percentages.

## Results

From a total of 3798 patients followed, 557 medication errors were identified, with a prevalence of 14.6%. Among 2025 males followed, 327 (16.14%) medication errors were identified, and among 1773 females followed, 230 (12.97%) medication errors were identified. The highest prevalence of medication errors was identified in patients in the age group 51-60 years with the prevalence of 3.76% followed by 41-50 years (2.71%) age group and 31-40 years (2.58%). Most of the medication errors were observed in patients receiving more than six medications with the prevalence of 6.71% and the least medication errors were observed among patients receiving one to three medications (3.34%). Further details and prevalence of medication errors are presented in Table 2.

**Table 2:** Demographics of the patients and related information (n = 3798) MEs: Medication errors

Characteristics	Group	Patients Followed (n = 3798)	MEs (n = 557 (%))	Prevalence (%)
Age (Years)	18-30	394	79 (20.05)	2.08
	31-40	407	98 (24.07)	2.58
	41-50	676	103 (15.23)	2.71
	51-60	1523	143 (9.38)	3.76
	61-70	451	75 (16.62)	1.97
	≥71	347	59 (17)	1.55
Gender	Male	2025	327 (16.14)	8.6
	Female	1773	230 (12.97)	6.05
Medications Received Per Patient	One to three	1055	127 (12.03)	3.34
	Four to Six	1285	175 (13.61)	4.6
	≥ Six	1458	255 (17.48)	6.71

Types of medication errors

Of 557 medication errors, 208 medication errors were prescription-related, with a prevalence of 5.47%, followed by 156 documentation errors (4.1%), administration errors (2.86%), and medication and dispensing errors (2.21%) (Table 3).

**Table 3:** Types of medication errors (n = 557) MEs: Medication errors

Sl. No.	Type of Medication Error	Number of MEs, n (%)	Prevalence
1	Prescribing	208 (37.34)	5.47
2	Documentation	156 (28)	4.10
3	Administration	109 (19.56)	2.86
4	Dispensing	84 (15.08)	2.21

These medication errors were further classified into subgroups to understand their nature (Table 3). Among the prescribing errors (208), illegible writing of prescription [85 (40.87%)] accounted for more medication errors followed by incomplete filling of prescription [57 (27%)]. The wrong dose of medication was found in 33 (15.8%) prescriptions, 22 (10.57%) prescriptions had error-prone abbreviations, and 11 (5.28%) of prescriptions had the wrong dosage form of medications (Table 4A).

Among 156 documentation errors, incorrect documentation of the name of the medication was found in 45 (28.84%) medical records, a missed signature of the respective staff after administration of medication was found in 42 (26.9%) medical records followed by wrong documentation of dose of medication [30 (19.23%)], missed documentation of the name of the medication in the treatment chart [21 (13.46%)] and transcription errors [18 (11.53%)] (Table 4B).

Among 109 administration errors, omission errors were the most common [41 (37.61%)] followed by extended use of medication [29 (26.6%)], wrong frequency of administration of medications [18 (16.5%)], wrong dose of medication administered [15 (13.76%)] and wrong medication administered [6 (5.5%)] (Table 4C).

Among 84 dispensing errors, 32 (38.1%) patients were dispensed the wrong medication followed by the wrong dosage of medication [28 (33.3%)] and wrong dose of medication [24 (28.57%)] (Table 4D). Table 4 also specifies the type of HCPs involved in these MEs.

**Table 4:** Subtypes of medication errors MEs: Medication errors; HCP: Healthcare professional.

A: Types of Prescribing Error (n = 208)			Type of HCP Involved in Prescribing Error, n (%)				
Sl. No.	Subtype	MEs, n (%)	Doctor	Postgraduate student	Intern	Pharmacist	Nurse
1	Illegible prescription	85 (40.87)	16 (18.82)	32 (27.64)	37 (43.52)	0	0
2	Incomplete prescription	57 (27.4)	12 (21.05)	24 (42.1)	21 (36.84)	0	0
3	Wrong dose of the medication	33 (15.86)	7 (21.21)	11 (33.33)	15 (45.45)	0	0
4	Error prone abbreviation	22 (10.57)	5 (22.72)	7 (31.81)	10 (45.45)	0	0
5	Wrong dosage form of the medication	11 (5.28)	0	5 (45.45)	6 (54.54)	0	0
B: Types of Documentation Error (n = 156)			Type of HCP Involved in Documentation Error n (%)				
Sl. No.	Subtype	MEs, n (%)	Doctor	Postgraduate student	Intern	Pharmacist	Nurse
1	Wrong documentation of name of the medication	45 (28.84)	10 (22.22)	28 (62.22)	7 (15.55)	0	0
2	No signature in treatment chart after administration of the medication	42 (26.92)	0	0	0	0	42 (100)
3	Wrong documentation of dose of the medication	30 (19.23)	2 (6.66)	13 (43.33)	15 (50)	0	0
4	Name of the medication was not mentioned in the treatment chart	21 (13.46)	0	12 (57.14)	9 (42.85)	0	0
5	Transcription	18 (11.53)	0	10	8	0	0
C: Types of Administration Error (n = 109)			Type of HCP Involved in Administration Error, n (%)				
Sl. No.	Subtype	MEs, n (%)	Doctor	Postgraduate student	Intern	Pharmacist	Nurse
1	Omission	41 (37.61)	0	0	0	0	41 (100)
2	Extended use of medication	29 (26.6)	0	0	0	0	29 (100)
3	Wrong frequency of medication	18 (16.51)	0	0	0	0	18 (100)
4	Wrong dose of medication	15 (13.76)	0	0	0	0	15 (100)
5	Wrong medication	6 (5.5)	0	0	0	0	6 (100)
D: Types of Dispensing Error (n = 84)			Type of HCP Involved in Dispensing Error, n (%)				
Sl. No.	Subtype	MEs, n (%)	Doctor	Postgraduate student	Intern	Pharmacist	Nurse
1	Wrong medication dispensed	32 (38.09)	0	0	0	32 (100)	0
2	Wrong dosage form of medication dispensed	28 (33.33)	0	0	0	28 (100)	0
3	Wrong dose of medication dispensed	24 (28.57)	0	0	0	24 (100)	0

Causes of medication errors

Apart from the types and subtypes of medication errors, where possible the causes of medication errors were identified in this study. Of these 557 medication errors, it was found that the majority of errors were due to perceived lack of time [213 (38.24%)], of which 166 (28.7%) errors were reported while writing the prescription and 47 (8.14%) during documentation in medical records. Work overload among participants accounted for 133 (23.87%) of total medication errors. Lack of knowledge of participants while writing the prescription, administration of medications, and dispensing of medications were reported to cause 62 (11.13%) medication errors. Change in the duty shift of HCPs accounted for 24 (4.15%) and 23 (3.98%) of documentation and administration errors, respectively. Look-alike and sound-alike (LASA) medications caused 10 (1.73%) dispensing errors. Further details of the causes of each medication errors are presented in Table 5.

**Table 5:** Causes of medication errors (n = 557) HCP: Healthcare professional

Causes of Medication Errors [n = 557 (%)]						Type of HCP Involved, n (%)				
Causes	Prescription	Documentation	Administration	Dispensing	Total	Doctor	Postgraduate student	Intern	Pharmacist	Nurse
Duty shift	0	24 (4.15)	23 (3.98)	0	47 (8.43)	1 (2.12)	11 (23.4)	12 (25.53)	0	23 (48.93)
Forgetfulness	0	0	31 (5.37)	8 (1.38)	39 (7)	0	3 (7.69)	2 (5.12)	3 (7.69)	31 (79.48)
Illegible documentation	0	0	3 (0.51)	0	3 (0.53)	0	0	0	0	3 (100)
Lack of attention	0	32 (5.54)	0	0	32 (5.74)	2 (6.25)	12 (37.5)	8 (25)	5 (15.62)	5 (15.62)
Lack of knowledge	42 (7.27)	0	7 (1.21)	13 (2.25)	62 (11.13)	5 (8.06)	15 (24.19)	23 (37.09)	12 (19.35)	7 (11.29)
Lack of time	166 (28.7)	47 (8.14)	0	0	213 (38.24)	34 (15.96)	75 (35.21)	53 (24.88)	2 (0.93)	49 (23)
Look alike Sound alike (LASA) medications	0	0	0	10 (1.73)	10 (1.79)	0	0	0	10 (100)	0
Too many prescriptions	0	0	0	11 (1.9)	11 (1.97)	0	0	0	11 (100)	0
Unable to read prescription	0	0	0	7 (1.21)	7 (1.25)	0	0	0	7 (100)	0
Work overload	0	53 (9.18)	45 (7.79)	35 (6.06)	133 (23.87)	10 (7.51)	26 (19.54)	30 (22.55)	34 (25.56)	33 (24.81)

Medications involved in medication errors

Of 557 medication errors, the majority involved the medications belonged to alimentary tract and metabolism (n = 167). Of these medications, pantoprazole was involved in 42 (7.54%) of medication errors, followed by ondansetron in 32 (5.74%), metformin in 29 (5.2%), multivitamins in 28 (5.02%), domperidone in 16 (2.87%), insulin in 11 (1.97%), and rabeprazole in nine (1.61%). Anti-infectives for systemic use accounted for 159 (28.54%) medication errors. Of these, the majority of errors involved piperacillin + tazobactam (49, 8.79%). Medications under the cardiovascular system were involved in 146 (26.21%) medication errors, followed by medications under systemic hormonal preparations (32, 5.74%), blood and blood forming organs (19, 3.41%), respiratory system (18, 3.23%), and nervous system (16, 2.87%). Further details of all medications with respective type medication errors are presented in Table 6.

**Table 6:** Anatomical Therapeutic Chemical Classification of medications (n = 557) MEs: Medication errors

ATC Classification	Name of the medication	Number of MEs, n (%)	Prescription (n = 208)	Documentation (n = 156)	Administration (n = 109)	Dispensing (n = 84)
Alimentary Tract and Metabolism (n = 167 (29.98%))	Pantoprazole	42 (7.54)	14	12	8	8
	Ondansetron	32 (5.74)	19	10	3	0
	Metformin	29 (5.2)	12	4	10	3
	Multivitamin	28 (5.02)	13	7	4	4
	Domperidone	16 (2.87)	2	12	0	2
	Insulin	11 (1.97)	6	2	2	1
	Rabeprazole	9 (1.61)	3	2	0	4
Anti-infectives for systemic use (n = 159 (28.54%))	Piperacillin + Tazobactam	49 (8.79)	21	13	7	8
	Ceftriaxone	38 (6.82)	15	19	2	2
	Cefuroxime	29 (5.2)	16	3	5	5
	Ciprofloxacin	24 (4.3)	3	4	7	10
	Azithromycin	19 (3.41)	12	0	4	3
Cardiovascular system (n = 146 (26.21%))	Atorvastatin	35 (6.28)	12	12	5	6
	Telmisartan	30 (5.38)	12	10	5	3
	Metoprolol	29 (5.2)	7	10	12	0
	Cilnidipine	23 (4.12)	7	6	5	5
	Furosemide	15 (2.69)	4	3	6	2
	Amlodipine	8 (1.43)	0	2	0	6
	Spironolactone	6 (1.07)	1	3	1	1
Systemic Hormonal Preparations (n = 32 (5.74%))	Methylprednisolone	13 (2.33)	7	0	5	1
	Dexamethasone	11 (1.97)	3	0	4	4
	Levothyroxine	8 (1.43)	0	7	0	1
Blood and Blood Forming Organs (n = 19 (3.41%))	Aspirin	10 (1.79)	4	2	3	1
	Clopidogrel	9 (1.61)	4	0	3	2
Respiratory System (n = 18 (3.23%))	Salbutamol + Ipratropium bromide	10 (1.79)	2	4	3	1
	Cetirizine	8 (1.43)	1	6	0	1
Nervous System (n = 16 (2.87%))	Phenytoin	9 (1.61)	5	3	1	0
	Levetiracetam	7 (1.25)	3	0	4	0

NCC MERP categorization of medication errors

The observed medication errors were categorized based on the NCC MERP categorization of medication errors (Table 7). It was found that the majority of medication errors belonged to category A [261 (46.85%)], followed by category B [143 (25.67%)], category C [118 (21.18%)], category D [23 (4.12%)], and category E [12 (2.15%)]. The majority of prescription (133/208) and documentation (105/156) errors were category A. Most administration errors belonged to category B (44/109) and the least number of administration medication errors were category E (12/109). Among dispensing errors, most of the medication errors belonged to category C (51/84) followed by category A (23/84) and category B (10/84).

**Table 7:** National Coordinating Council for Medication Error Reporting and Prevention (NCC MERP) categorization of medication errors Categories: A: Circumstances or events that have the capacity to cause an error B: An error occurred, but the medication did not reach the patient (error, no harm) C: An error occurred that reached the patient but did not cause potential harm (error, no harm) D: An error occurred that resulted in the need for increased patient monitoring but no patient harm (error, no harm) E: An error occurred that resulted in the need for treatment or intervention and caused temporary patient harm (error, harm)

Types of Medication Error	Category A	Category B	Category C	Category D	Category E
Prescription (n = 208)	133	52	23	0	0
Documentation (n = 156)	105	37	14	0	0
Administration (n = 109)	0	44	30	23	12
Dispensing (n = 84)	23	10	51	0	0
Total (n = 557)	261 (46.85%)	143 (25.67%)	118 (21.18%)	23 (4.12%)	12 (2.15%)

## Discussion

The present study focused on identifying and analyzing the medication errors in a tertiary care hospital during the study period. We found that the incidence of MEs was 1.5 per 100 prescriptions. This was very similar to other studies [5, 10]. The most common type of MEs encountered in our study was a prescription error which was also seen in other studies [6, 17]. Of the prescribing errors, illegible and incomplete prescriptions were seen in the majority of the cases. This subtype of error is a fairly common source of ME [18, 19]. The major factors plaguing prescription errors in our study were either lack of knowledge most often by medical interns or time contributed mostly by postgraduate medical students.

Additionally, it is recommended that the HCP uses capital letters while mentioning the name of the medications and clear legible handwriting to complete the prescription. Alternately, the use of computerized physician order entry (CPOE) can reduce prescribing errors and increase adherence to complete a prescription [20].

Consistent with earlier research findings, the prevalence of documentation error was found to be 28% [6,11]. Incorrect documentation of the name of the medication and wrong dose documentation was the major reason for transcription error. In our study setting, documentation was largely being taken care of by junior doctors. Their lack of experience and inadequate knowledge of proper documentation may have contributed to this error. There needs to be proper training directed towards a systematic approach to medical transcription and documentation. It should be noted that most of the documentation errors were prevented before they could reach the patient. This was done by the intervention of clinical pharmacists and consultant doctors.

Administration error was found in 20% of the cases of which omission followed by extended use and incorrect frequency of drug was the most common subtype. These administration errors can be prevented with effective communication between physicians and nurses [21]. Additionally, the experience and knowledge of a nursing team were found to have a linear relationship with MEs [22]. Clinical pharmacists can also play a vital role in preventing administration errors by cross-verification and highlighting dosage and duration on the medication chart.

Dispensing errors accounted for 15% of the total errors. The most common subtype being the incorrect form/dose of drug dispensed. Hospitals can cut down significantly on dispensing errors by using automated dispensing cabinets, barcode medication administration, and closed-loop electronic medication management systems as demonstrated by a systematic review by Zheng et al. [23].

When analyzing for the causes of MEs, we observed that lack of time and the increased workload was the most prevalent contributor. In our study setting, both nurses and doctors in each ward were consistently understaffed leading to increased workload, stressful work environment, higher chances of mistake in drug admission and causes of burnout syndromes [24]. Studies show that the required ratios of nurses and doctors in India are lower when compared with the nationally recommended ratios i.e., 1:6 for nurse-to-patient ratio and 1:1000 for the doctor to population ratio [25, 26]. Furthermore, we found that one in 10 MEs was caused during shift changes. We need to adopt a standardized procedure for shift change that can help eliminate such errors.

Apart from the types of medication errors, we identified medications involved in medication errors and found that piperacillin + tazobactam, ceftriaxone, and pantoprazole were the most common medications involved. This can be explained by a fact that antibiotics and proton pump inhibitors are known to be overprescribed regularly due to their use as prophylaxis because of uncertainty in the diagnosis [27, 28]. We need to make sure that physicians adhere to the latest therapeutic guidelines when prescribing medications to avoid such errors.

It was found that the majority of medication errors were in category A of the severity of medication errors. These findings were similar to a study conducted by Thomas et al. [29] where 77.3% of medication errors belonged to category A. Besides, around 6% of all reported medication errors in this study were in either category D or category E which required monitoring of the patient. The frequency of these critical medication errors can be minimized by sensitizing healthcare professionals on various types and causes of medication errors along with their preventive measures through regular training sessions.

The study was conducted for a period of almost a year. We promised to maintain the confidentiality of the healthcare professionals involved and the non-punitive reporting culture implemented in this study had encouraged healthcare professionals to report medication errors. There were a few limitations in the study like this was a single-center study, only the general medicine in-patient wards were included. Another limitation is that only medication errors were reported while monitoring error was not reported and evaluated. The causes for reported medication errors were subjective which may have altered the outcomes of the study.

## Conclusions

Overall, lack of time, work overload, lack of knowledge on the administration of medications were common causes mentioned by participants for reported medication errors. Antibiotics and proton pump inhibitors were the most common drugs involved in MEs. Prescribing and documentation errors were most prevalent. Implementation of systems like strict adherence to treatment guidelines, computerized provider order entry (CPOE), barcode medication administration, and closed-loop electronic medication management systems may greatly help reduce MEs. Even though a majority of MEs did not harm the patient, we need to take a systematic approach in documentation and prevention of MEs employing adequate training to healthcare professionals, and implementing routine medication audits.
